# CDK4/6 inhibition as maintenance and combination therapy for high grade serous ovarian cancer

**DOI:** 10.18632/oncotarget.24585

**Published:** 2018-02-26

**Authors:** Mangala Iyengar, Patrick O’Hayer, Alex Cole, Tara Sebastian, Kun Yang, Lan Coffman, Ronald J. Buckanovich

**Affiliations:** ^1^ University of Michigan, Department of Cellular and Molecular Biology, Ann Arbor, MI 48109, USA; ^2^ University of Michigan, Medical Scientist Training Program, Ann Arbor, MI 48109, USA; ^3^ University of Michigan, School of Literature, Science and the Arts, Ann Arbor, MI 48109, USA; ^4^ University of Michigan, Division of Hematology and Oncology, Department of Internal Medicine, Ann Arbor, MI 48109, USA; ^5^ Magee Women’s Research Institute, University of Pittsburgh, Department of Internal Medicine, Pittsburgh, PA 15213, USA

**Keywords:** ovarian cancer, Ribociclib, CDK 4/6 inhibitor, chemotherapy resistance, cell cycle

## Abstract

High grade serous ovarian cancer (HGSOC) is a disease with a high relapse rate and poor overall survival despite good initial responses to platinum-based therapy. Cell cycle inhibition with targeted CDK4/6 inhibitors is a new therapeutic approach showing promise as a maintenance therapy in cancer. As multiple genes in the CDK4/6 pathway are commonly mutated or dysregulated in ovarian cancer, we evaluated the efficacy of the CDK4/6 inhibitor Ribociclib alone, in combination with chemotherapy, and as maintenance therapy in several models of HGSOC. Ribociclib restricted cellular proliferation in multiple ovarian cancer cell lines. Restricted proliferation was associated with a pseudo-senescent cellular phenotype; Ribociclib-treated cells expressed markers of senescence, but could rapidly re-enter the cell cycle with discontinuation of therapy. Surprisingly, concurrent Ribociclib and cisplatin therapy followed by Ribociclib maintenance was synergistic. Evaluation of the cell cycle suggested that Ribociclib may also act at the G2/M check point via dephosphorylation of ATR and CHK1. Consistent with this mechanism, Ribociclib demonstrated clear activity in both platinum-resistant and platinum-sensitive tumor models *in vivo*. This work supports clinical trials using Ribociclib in combination with cisplatin and as a maintenance therapy in ovarian cancer.

## INTRODUCTION

High grade serous ovarian cancer (HGSOC) is the most lethal gynecological cancer in the United States and is characterized by a high recurrence rate [1]; 70% of patients relapse and succumb to their disease despite initially successful chemotherapy. This is largely because most patients present with disseminated disease (stages III/IV) at diagnosis [2]. Patients with recurrent ovarian cancer inevitably develop resistance to standard platinum-based chemotherapy and additional chemotherapy does not offer significant survival benefit [3]. Therefore, non-cytotoxic maintenance therapies which could extend disease-free survival may improve a patient’s quality of life and potentially prolong survival.

CDK4/6 inhibition is an emerging cytostatic therapy targeting cell cycle progression. A heterotrimeric complex of Cyclin D1, CDK4, and CDK6 is required to phosphorylate RB1, which eventually leads to the transcription of genes required for S phase. Therefore, CDK4/6 inhibition blocks the G1-S phase transition, forcing G1 arrest (reviewed in [4]). CDK4/6 inhibitors have shown promise in many tumors *in vitro*, such as neuroblastoma [5], liposarcoma [6], breast cancer [7], mantle cell lymphoma [8], non-small cell lung cancer [9], and germ cell tumors [10]. Importantly, there are also positive clinical results in patients. In metastatic breast cancer, Palbociclib in combination with letrozole doubled progression-free survival from 10 to 20 months compared to letrozole alone in a Phase II trial [11] and from 19.3 to 30.4 months in a Phase III trial [12]. Similarly, Ribociclib showed a significant impact in breast cancer [13], and both Palbociclib and Ribociclib are now FDA-approved in combination with an aromatase inhibitor as frontline treatment in ER^+^/HER2^–^ metastatic breast cancer.

In ovarian cancer, CDK4/6 inhibitors have shown promise *in vitro*. Response to CDK4/6 inhibitors has been linked to the mutational status of p16 and Rb [14]. CDK4/6 inhibitors have also been linked to targeting cancer stem cells [15]. However, resistance mechanisms have also been reported [16]. Clinical data regarding CDK4/6 inhibition in ovarian cancer are sparse. While Phase 1 (NCT03294694, NCT02897375) and Phase 2 (NCT02657928) trials of CDK4/6 inhibition in combination with other therapies in ovarian cancer have recently opened, no data is available yet from these studies. However, the CDK4/6 inhibitor abemaciclib as monotherapy did produce stable disease in two patients and a CA-125 response in a third patient [17]. Importantly, CDK4/6 inhibitors have generally been well-tolerated and side effects have been successfully managed by dose-reduction [18]. Therefore, these compounds could be useful as maintenance therapies or combination therapies in patients with chemotherapy-resistant disease.

We mined the TCGA database [19] and found that ∼40% of patients with HGSOC have mutations/dysregulation of various genes which regulate the G1 to S phase cell cycle transition, which is consistent with previous literature (reviewed in [20]) . We therefore tested CDK4/6 inhibition with Ribociclib both *in vitro* and *in vivo* and demonstrated a significant delay in ovarian cancer cell growth via the induction of a pseudo-senescent state. The combination of Ribociclib and cisplatin led to growth-arrest *in vitro* and significantly delayed tumor growth *in vivo*; therefore, Ribociclib appears to be a promising therapeutic for ovarian cancer treatment.

## RESULTS

### Mutations and dysregulation of genes in the CDK4/6 pathway are common in ovarian cancer

**Figure 1:**
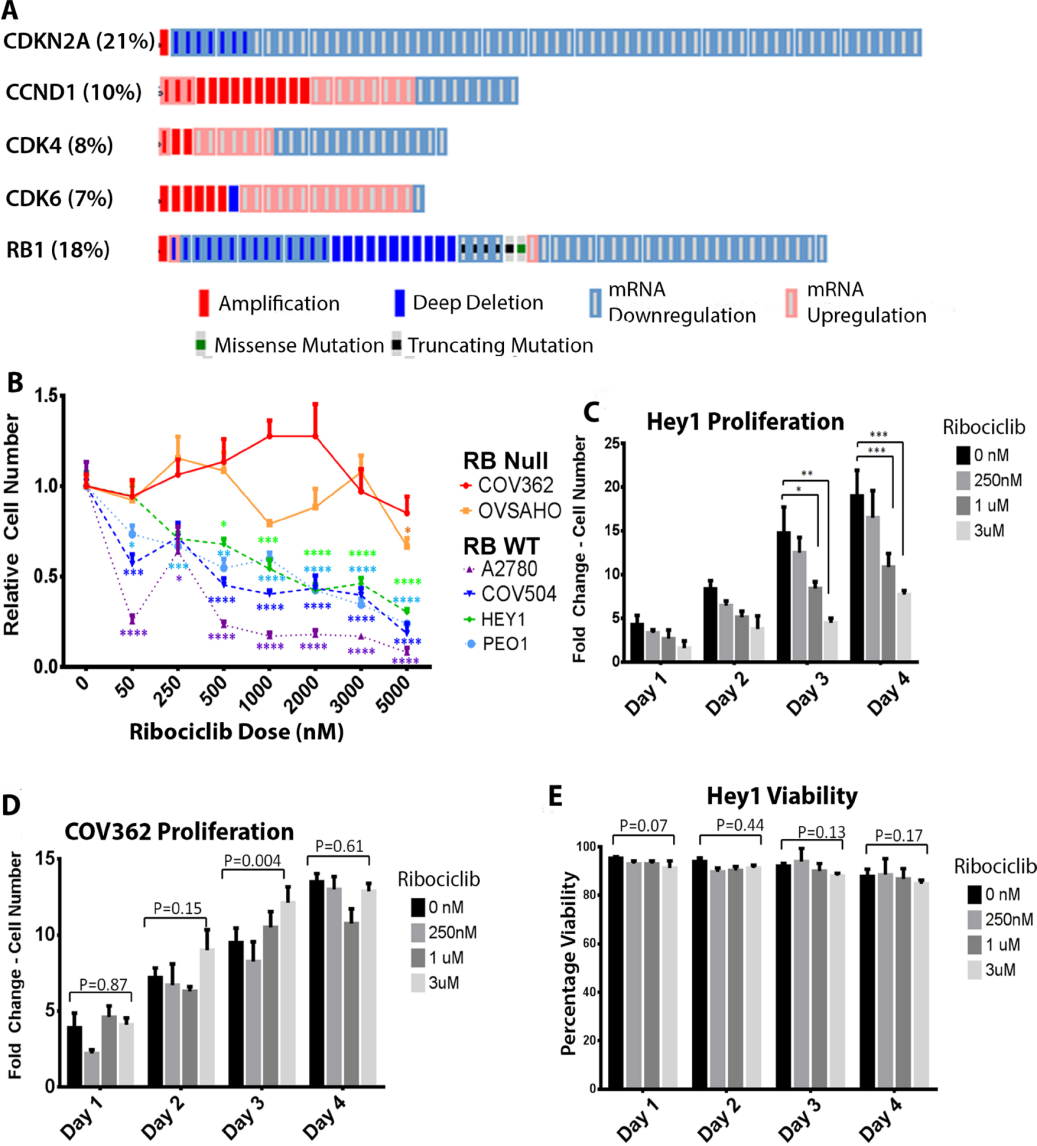
Ribociclib is a rational target in ovarian cancer (**A**) Analysis of 316 tumors from the TCGA database showing mutations and mRNA dysregulation of genes known to regulate the G1-S phase transition. (**B**) Cell numbers as a proportion of untreated control cell numbers in the indicated cell lines after 72 hours of treatment with the indicated doses of Ribociclib. (**C**) Fold-change in cell number over time in Hey1 cells (Rb^WT^) treated with the indicated doses of Ribociclib. (**D**) Fold-change in cell number over time in COV362 cells (Rb^null^) treated with the indicated doses of Ribociclib. (**E**) Analysis of cellular viability in Hey1 cells treated with the indicated doses of Ribociclib. All samples were analyzed at least in triplicate with each experiment performed three times. ^*^p < 0.05, ^**^p < 0.01, ^***^p < 0.001, ^****^p<0.0001 by two-sample, two-tailed *t*-tests comparing the indicated values in C and D and one-way ANOVA comparing groups in B.

The cBioPortal browser was used to mine data from The Cancer Genome Atlas (TCGA, [19]) to perform mutational analysis of genes in the CDK4/6 pathway. Mutations and significant dysregulation of mRNA expression (z-score < –2 or >2) were common in patients with HGSOC (Figure 1A). CDKN2A (also known as p16^INK4a^) is a tumor suppressor that normally serves as a brake on cell cycle progression by inhibiting CDK4 and CDK6 [21]. Interestingly, 21% of ovarian cancer patients showed CDKN2A deletions or significant downregulation. Another 16% showed significant amplifications or increases in mRNA expression of CDK4, CDK6, and/or Cyclin D1 expression; both these classes of mutations would contribute to an aberrantly overactive cell cycle and, presumably, tumor growth. Of note, RB1 was deleted or significantly downregulated in 17% of HGSOC patients; these patients may be less likely to respond to CDK4/6 inhibition. Overall, our data (Figure 1A) show that there is a large subset of HGSOC patients who would likely benefit from therapy with a CDK4/6 inhibitor. Therefore, we investigated the CDK4/6 inhibitor Ribociclib (LEE-011)(Novartis) in HGSOC.

### Ribociclib affects growth in multiple ovarian cancer cell lines

A2780, Hey1, COV362, COV504, PEO1, and OVSAHO ovarian cancer cell lines were treated with increasing doses of Ribociclib for 3 days as detailed in Figure 1B and cell proliferation was quantified by cell counts with trypan blue exclusion. The RB1^WT^ cell lines A2780, Hey1, COV504, and PEO1 showed dose-dependent growth inhibition (Figure 1B). As RB1 is a core downstream target of CDK4 and CDK6, RB1^null^ cells should be resistant to CDK4/6 inhibition. To verify on-target effects, the RB1^null^ lines COV362 and OVSAHO were also treated with Ribociclib and were unresponsive (Figure 1B). For more detailed analysis, we treated the Rb^WT^ cell line Hey1 and the Rb^null^ line COV362 with Ribociclib and analyzed cell counts and viability daily. Relative to control treatment, Ribociclib decreased the number of Hey1 cells in a dose-dependent manner (Figure 1C) without affecting Hey1 cell viability (Figure 1E), suggesting that treatment leads to growth-arrest rather than cell death in this RB^WT^ cell line. However, Ribociclib did not affect Rb^null^ COV362 cell proliferation (Figure 1D) or viability (Supplementary Figure 1A) even at the highest doses, suggesting that decreased proliferation is an on-target effect of Ribociclib.

### Ribociclib decreases cell proliferation by arresting cells in G1 in a ‘pseudo-senescent’ state

**Figure 2:**
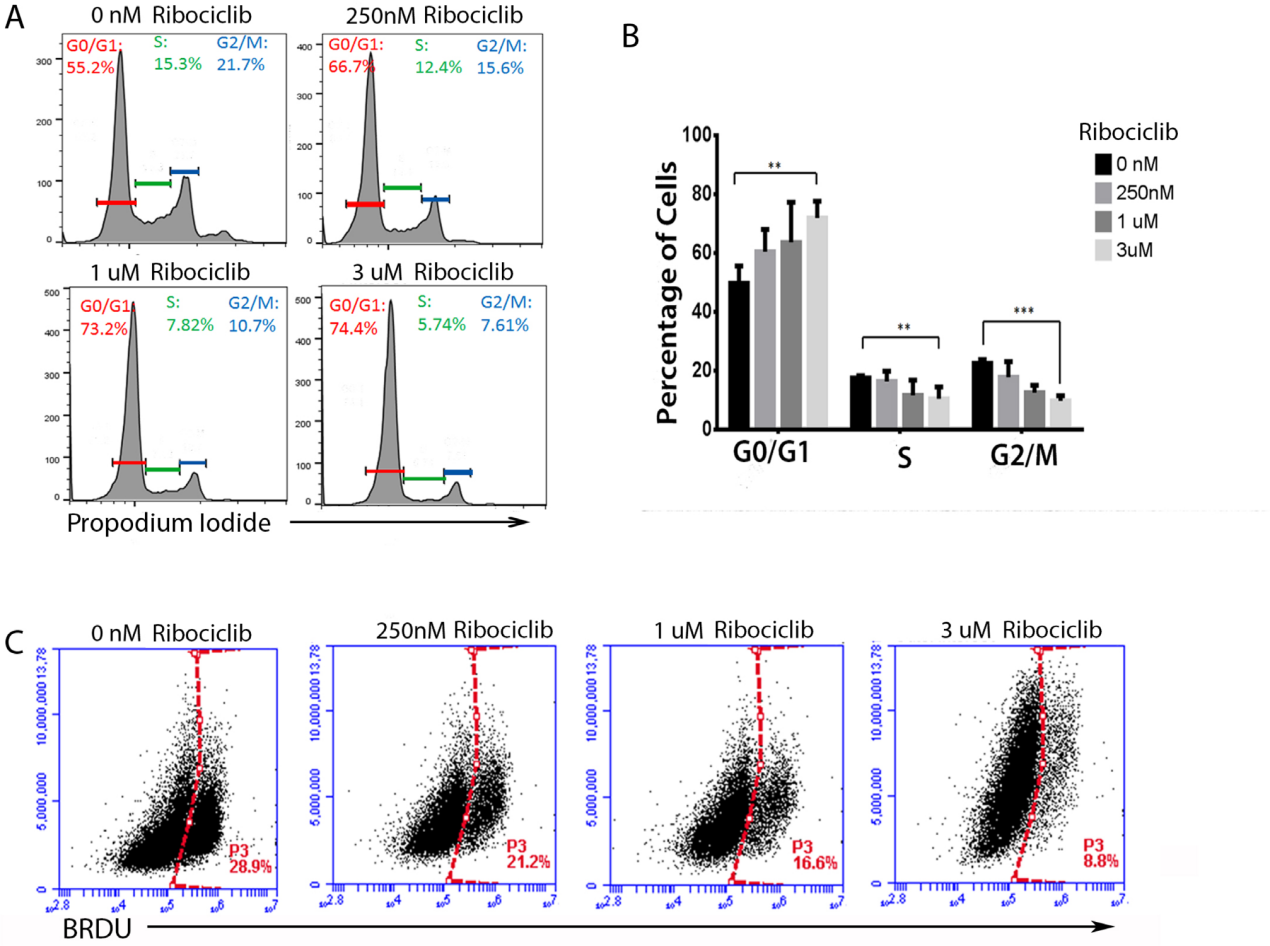
Ribociclib treatment leads to G1 arrest and decreased BrdU incorporation (**A**) Representative cell cycle profiles of Hey1 ovarian cancer cells treated with Ribociclib for 72 h and (**B**) summary of cell cycle phase shifts. (**C**) BrdU incorporation in Hey1 ovarian cancer cells after 72 h of treatment with the indicated doses of Ribociclib. All samples were analyzed in triplicate with each experiment replicated at least once. FACS samples counted at least 10,000 events. ^*^p < 0.05, ^**^ *p* < 0.01, ^***^p < 0.001 by two-sample, two-sided *t*-test.

Cell cycle phase analysis with propidium iodide showed that Ribociclib treatment led to a dose-dependent accumulation of Hey1 cells in the G1/G0 phase of the cell cycle, with a concomitant decrease in the number of cells in the S and G2/M phases (Figure 2A–2B). This is consistent with the known role of CDK4 and CDK6 in regulating the G1-S transition [4]. We also observed a decrease in BrdU incorporation in Hey1 ovarian cancer cells during this treatment (Figure 2C), confirming a decrease in proliferation. The Rb^null^ line COV362 showed no cell cycle changes in response to Ribociclib, regardless of dose (Supplementary Figure 1B).

**Figure 3:**
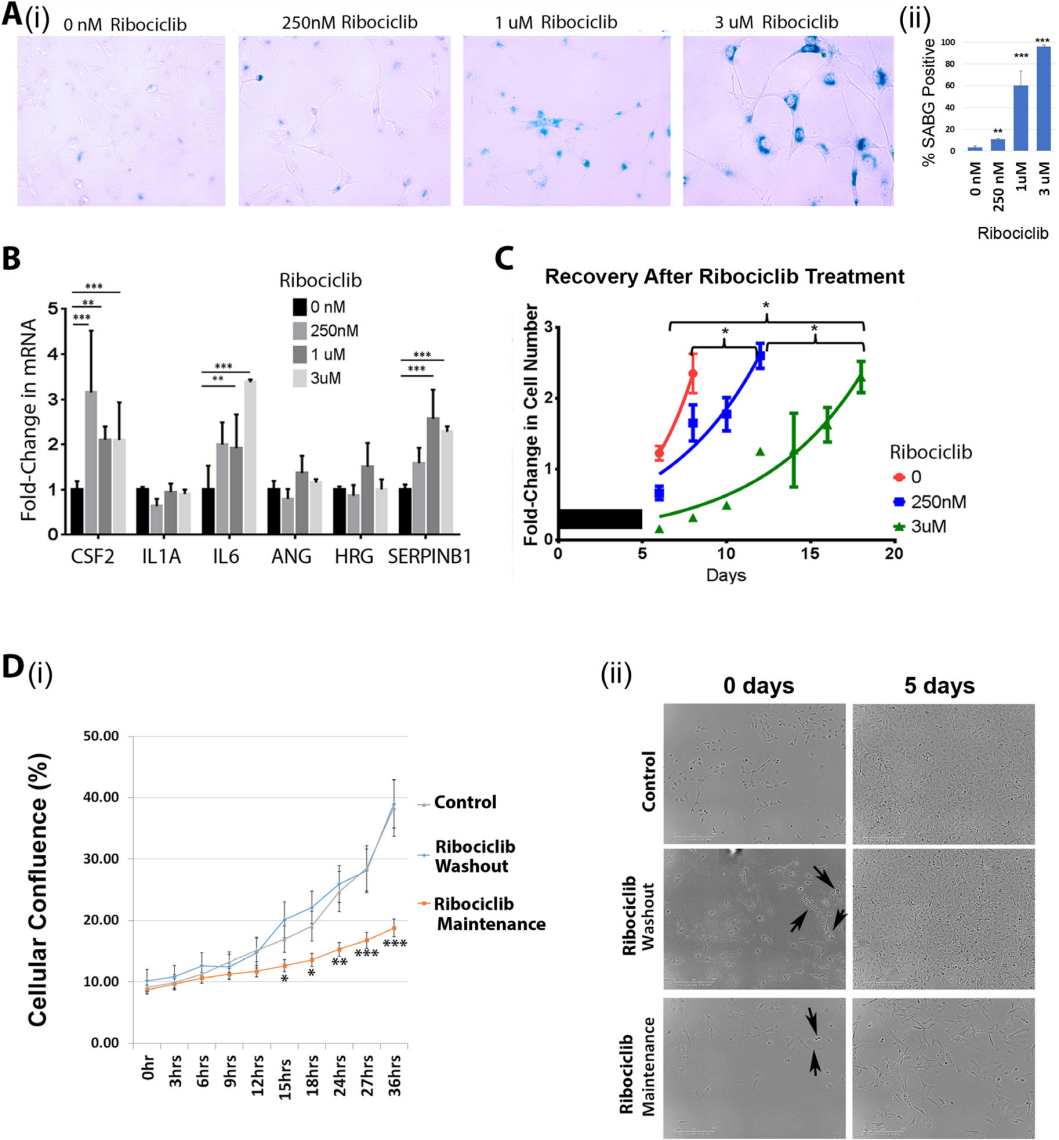
Ribociclib induces a pseudosenescent state in ovarian cancer cells (**A**) Senescence-associated β-galactosidase staining (i) and quantification (ii) following a 72 h treatment of Hey1 cells with the indicated dose of Ribociclib. (**B**) qRT-PCR analysis of mRNA expression of senescence-associated secretory genes after a 72 h treatment with the indicated dose of Ribociclib. (**C**) Fold-change in cell numbers of Hey1 cells after 5 days of treatment with the indicated dose of Ribociclib or vehicle (black bar) followed by discontinuation of treatment. (**D**)(i) Graph of confluence of control cells and cells treated continuously with Ribociclib for 5 days and then either maintained on therapy or washed and given fresh media 24 hours prior to monitoring. (ii) Representative photomicrographs from representative wells of the indicated treatment conditions in panel (i) at the start and end of imaging. Arrows indicate cells observed to be dividing. All samples were analyzed in triplicate with each experiment replicated at least once. ^*^p < 0.05, ^**^p < 0.01, ^***^p < 0.001 by two-sample, two-sided *t*-tests.

CDK4/6 inhibitors have been reported to induce senescence in cancer cells [5, 22]. We therefore evaluated the expression of Senescence Associated β-Galactosidase (SAβG) with Ribociclib treatment. Consistent with prior reports, we observed a clear increase in SAβG staining in treated Hey1 cells with increasing concentrations of Ribociclib (Figure 3Ai), with >95% of cells showing strong SAβG staining after three days of treatment (Figure 3Aii). Senescent cells are also reported to increase expression of numerous secretory proteins including CSF2, IL1A, IL6, ANG, HRG, and SERPINB1 (reviewed in [23]). However, Ribociclib treatment of Hey1 cells led to mRNA induction in only three of the six selected genes encoding senescence associated secretory proteins (Figure 3B).

Truly senescent cells are believed to permanently exit the cell cycle and should be unable to resume proliferation [24]. Even though cells treated with high-dose Ribociclib for 3 days demonstrate >95% SABG staining (Figure 3Ai-ii), cells demonstrate a stable albeit slow proliferation rate during treatment (Figure 1C). In addition, cells treated with Ribociclib for 5 days and then allowed to grow without the drug (termed “recovery”) resumed cycling after the drug washout, indicating a lack of true senescence (Figure 3C). Continued cell growth could be related to either a subpopulation of resistant cells or slower proliferation in the majority of cells. To evaluate this, we performed time lapse microscopy of (i) control cells, (ii) cells treated with high dose Ribociclib (3 µM) for 5 days followed by drug washout (incubation without drug in control growth medium), or (iii) cells with continuous Ribociclib (3 µM) treatment. After normalization for cell numbers, control cells and cells treated with Ribociclib followed by washout demonstrated similar proliferation rates (Figure 3Di). Image analysis confirmed proliferation of >70% of cells in each group, demonstrating that most cells can resume proliferation following Ribociclib washout. Furthermore, image analysis of cells maintained in continuous Ribociclib treatment demonstrated that >70% of cells were actively proliferating, but at a slower rate compared to the control and Ribociclib washout groups (Figure 3Dii). Therefore, it appears that rather than rapid proliferation of a small, resistant subpopulation, most cells continue to cycle at a slow rate when treated with Ribociclib, rather than entering a state of complete growth arrest. Together, these data suggest that CDK4/6 inhibition does not induce a truly senescent state in ovarian cancer cells.

### Ribociclib potentiates the impact of cisplatin

**Figure 4:**
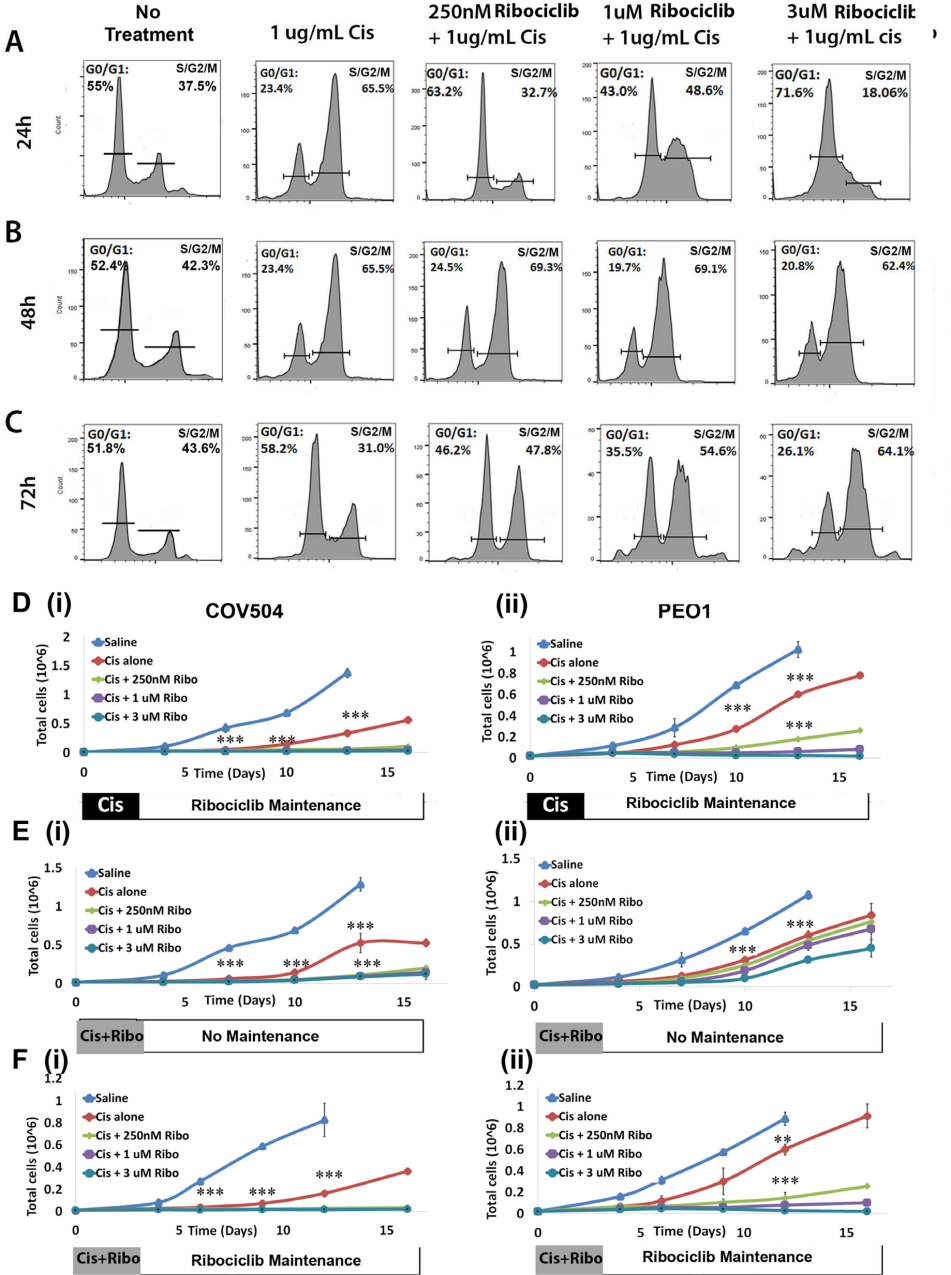
Ribociclib enhances cisplatin-induced G2/M arrest Cell cycle phase diagrams after 24 h (**A**), 48 h (**B**), and 72 h (**C**) of treatment with cisplatin alone or concurrent 1ug/mL cisplatin and the indicated dose of Ribociclib, or no treatment. (**D**) Absolute cell counts in (i) COV504 and (ii) PEO cells after treatment with cisplatin followed by maintenance Ribociclib. (**E**) Absolute cell counts in (i) COV504 and (ii) PEO cells after concurrent treatment with cisplatin and Ribociclib without maintenance Ribociclib. (**F**) Absolute cell counts in (i) COV504 and (ii) PEO1 cells after concurrent treatment with cisplatin and Ribociclib followed by maintenance Ribociclib. All samples were analyzed in triplicate with each experiment replicated at least once. ^*^p < 0.05, ^**^p < 0.01, ^***^p < 0.001 by two-sided, two-tailed *t*-tests.

Platinum-based chemotherapy is the standard of care for first-line treatment in ovarian cancer. We therefore investigated the combined impact of treatment with Ribociclib and cisplatin. We treated Hey1 ovarian cancer cells with 1ug/mL cisplatin alone or cisplatin in combination with Ribociclib (0 nM, 250 nM, 1 uM, or 3 uM). Consistent with previous reports and the known mechanism of cisplatin-induced DNA damage preventing cell cycle progression [25, 26], we observed that cisplatin treatment led to accumulation of cells in the S/G2/M phases of the cell cycle 24–48 hours after treatment (Figure 4A–4B). Cells treated with cisplatin alone recovered, with normalization of the cell cycle in surviving cells at 72 hours (Figure 4C). In contrast, the addition of Ribociclib to cisplatin significantly decreased the ability of cancer cells to move past the G2/M restriction point and back into a normal cell cycling pattern in a dose-dependent manner (Figure 4A–4C). 72 hours after treatment, the majority of cells treated with cisplatin and 3 uM Ribociclib remained in the G2/M peak (Figure 4C).

Given these results, we next used MTT assays to quantify the effects of Ribociclib on absolute and relative cell numbers remaining after cisplatin chemotherapy. Concurrent treatment with Ribociclib and cisplatin for 72 hours led to a decrease in the absolute number of surviving Hey1 cells (Supplementary Figure 2Ai). Normalization for the impact of Ribociclib on cell proliferation demonstrated a similar rate of cellular kill (Supplementary Figure 2Bi). Conversely, while pre-treatment with Ribociclib for 24 hours before chemotherapy led to a decrease in the absolute number of cells (Supplementary Figure 2Aii), normalization of cell numbers to adjust for cell number decrease related to Ribociclib exposure suggested that cell cycle arrest with Ribociclib prior to cisplatin exposure resulted in a higher proportion of surviving cells (Supplementary Figure 2Bii). When pre-treatment with Ribociclib was followed by a 24 hr washout period before cisplatin treatment, this effect disappeared (Supplementary Figure 2Aiii, 2Biii).

We further evaluated the timing of therapy and the addition of Ribociclib maintenance therapy using cancer cell recovery assays. Ribociclib maintenance therapy (initiated 72 hrs after initial treatment with cisplatin), potently synergized with cisplatin in the COV504, PEO1, and Hey1 ovarian cancer cell lines; in fact, control cells recovered effectively after cisplatin therapy, while cells treated with 1 uM or 3 uM Ribociclib as maintenance after cisplatin therapy remained unable to proliferate throughout the two-week observation period (Figure 4D; Supplementary Figure 3A). Combination indices for TD50 doses of cisplatin and doses of Ribociclib 250 nm–3 uM ranged from 0.2–0.39. Co-treatment for 3 days with Ribociclib and cisplatin, followed by no maintenance therapy (Figure 4E; Supplementary Figure 3C) effectively delayed cell growth, but the Hey1 and PEO1 cells resumed proliferation in the absence of continued Ribociclib treatment. Continued therapy with 1 uM or 3 uM Ribociclib effectively prevented cells from proliferating (Figure 4F; Supplementary Figure 3B). As seen in the MTT assays, pretreatment of cells with Ribociclib prior to cisplatin therapy was not an effective therapeutic regimen (Supplementary Figure 3D).

### Ribociclib and Ribociclib + Cisplatin treatment decreases pCHK1

**Figure 5:**
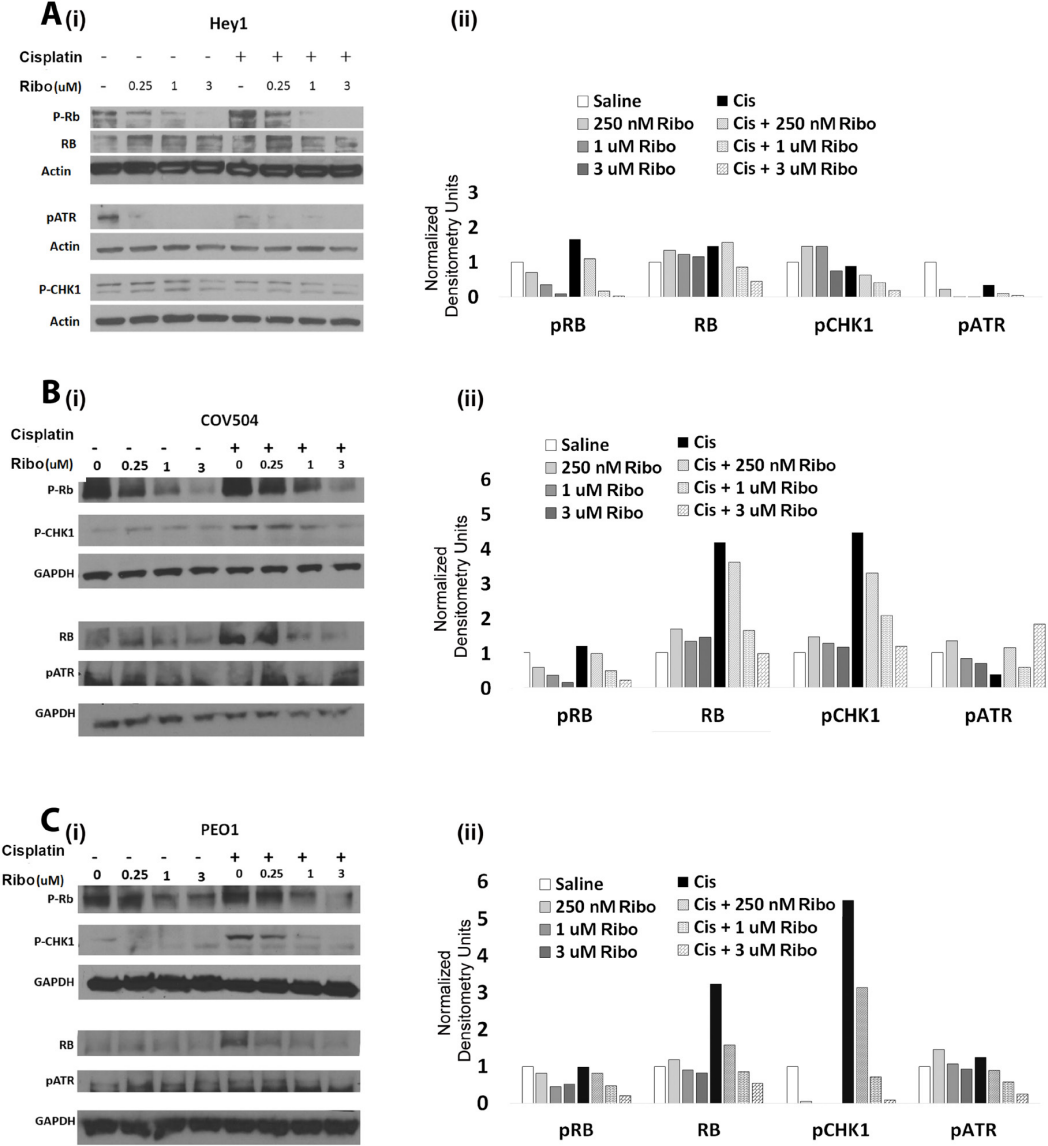
Ribociclib decreases pRb, ATR, and Chk1 alone and in combination with cisplatin (i) Western blot evaluation and (ii) densitometric quantification of total Rb, p-Rb, p-Chk1, and p-ATR in Hey1 (**A**), COV504 (**B**), and PEO1 (**C**) cells after 72 hours of the indicated doses of Ribociclib or a single dose of cisplatin followed by three days of Ribociclib. Gels have been cropped for clarity. All samples were analyzed in triplicate with each experiment replicated at least once for each cell line.

To verify on-target activity of Ribociclib, Hey1, COV504, and PEO1 cells were treated with increasing doses of Ribociclib either alone or combined with cisplatin, and lysates were collected for Western blot analysis of p-Rb. As predicted, Ribociclib treatment resulted in dose-dependent inhibition of Rb phosphorylation (Figure 5A–5C). Interestingly, co-treatment with Ribociclib and cisplatin decreased both pRB and total RB.

The potentiation of the impact of cisplatin with extended arrest of cells in the G2/M phase of the cell cycle data, as seen in Figure 4, suggests that CDK4/6 inhibition may have an unappreciated impact on the DNA damage response. We therefore evaluated the impact of Ribociclib on pATR and pChk1, which are known to participate in the DNA damage response. We found that in all three tested cell lines, Ribociclib decreased p-Chk1 in a dose dependent manner in the presence of cisplatin (Figure 5A–5C). pATR was similarly decreased in Hey1 cells (Figure 5A).

### Ribociclib is effective alone and in combination with cisplatin *in vivo*

**Figure 6:**
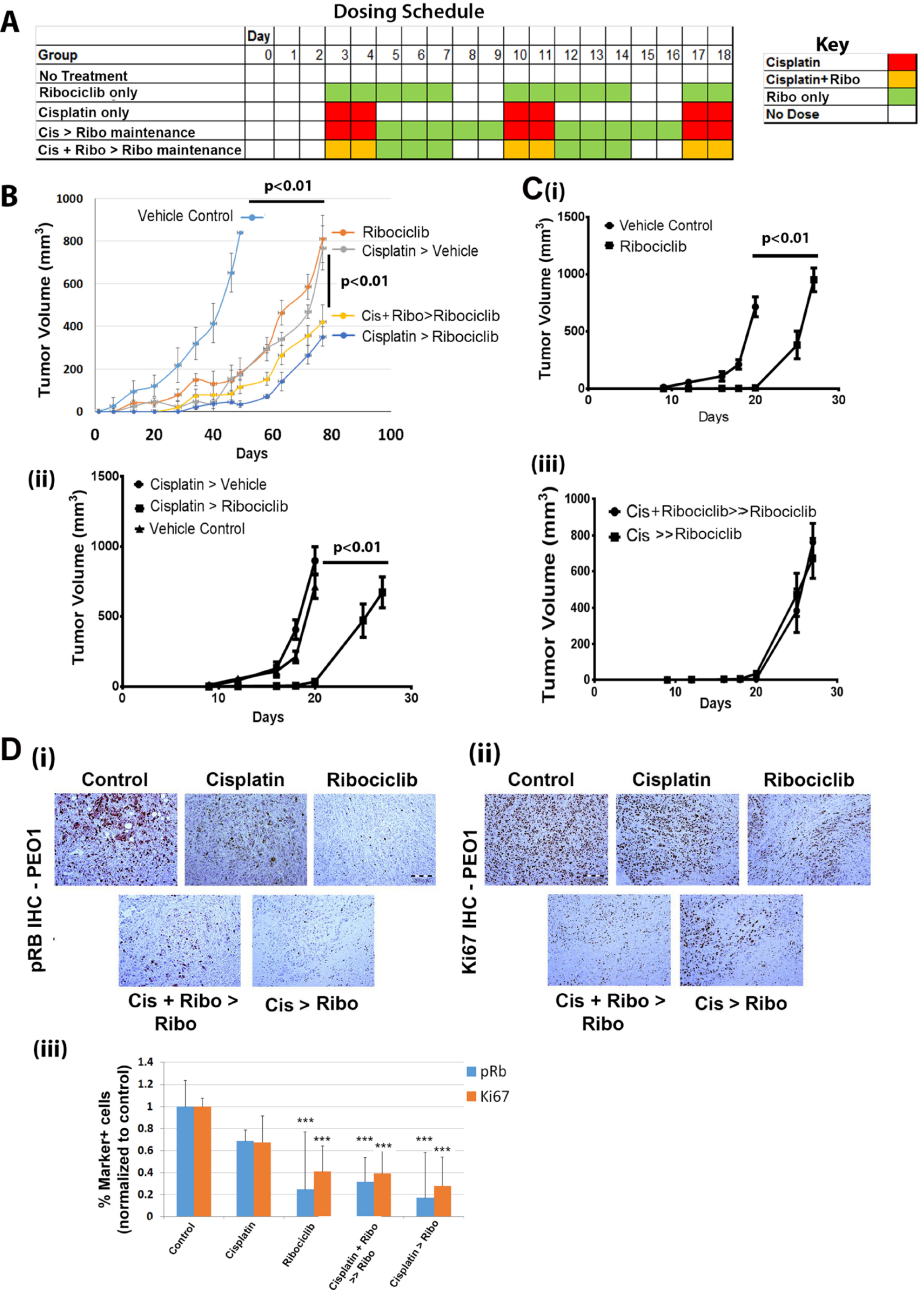
Ribociclib decreases Hey1 and PEO1 ovarian cancer tumor xenograft growth in combination with cisplatin (**A**) Sample dosing schedule for Ribociclib-only maintenance therapy and various combinations of sequential and concurrent treatment with cisplatin and Ribociclib. (**B**) Tumor growth in platinum-sensitive PEO1 xenografts treated with the indicated combinations of cisplatin with or without concurrent Ribociclib treatment and with or without Ribociclib maintenance therapy. (**C**) Hey1 tumor xenograft growth when treated with (i) vehicle vs. Ribociclib, (ii) vehicle control vs. cisplatin followed by vehicle (Cisplatin > vehicle) vs. cisplatin followed by Ribociclib maintenance (Cisplatin > Ribociclib), or (iii) cisplatin concurrent with Ribociclib followed by Ribociclib maintenance (Cis + Ribociclib > Ribociclib) vs. cisplatin alone followed by Ribociclib maintenance (Cis > Ribociclib). (**D**) IHC analysis of (i) pRB and (ii) Ki67 in tumors from the indicated treatment groups, and (iii) quantification of pRB and Ki67 marker-positive cells in the in the indicated treatment groups. Five high power fields from three sections of three tumors in each group were scored. ^***^p < 0.001 relative to the control.

We next evaluated Ribociclib activity *in vivo* using platinum-sensitive PEO1 cell line xenografts. Ribociclib treatment (5 days on + 2 days off, as described in the dosing schedule in Figure 6A) was started three days after tumor initiation. Ribociclib treatment was as effective as cisplatin in slowing tumor growth in the PEO1 xenografts (Figure 6B). Cisplatin treatment, either as a single agent or concurrent with Ribociclib, followed by maintenance with Ribociclib, further restricted disease growth (Figure 6B; *p* < 0.01). No clear benefit of concurrent versus sequential therapy with cisplatin and Ribociclib was observed.

As platinum-resistance is an important clinical problem, we next evaluated the impact of single agent Ribociclib in the platinum-resistant Hey1 cell line. Compared to vehicle treatment, treatment with Ribociclib significantly delayed tumor growth (*p* < 0.01) (Figure 6Ci). Then, we tested the impact of Ribociclib as a maintenance therapy following cisplatin in Hey1 cells. Dosing schedules were established such that all treatment groups received two doses of cisplatin weekly and five doses of Ribociclib weekly (Figure 6A). The addition of Ribociclib maintenance therapy after cisplatin resulted in a ∼40% increase in time to tumor endpoint (defined as a total tumor burden >2,000 mg per mouse, >10% weight loss, tumor ulceration, or poor health of the animal) (Figure 6Cii). We also evaluated the impact of concurrent cisplatin+Ribociclib followed by Ribociclib maintenance vs. cisplatin alone followed by Ribociclib maintenance therapy. In this platinum-resistant cell line, there was no additional benefit of concurrent therapy versus maintenance alone (Figure 6Ciii).

Finally, we performed immunohistochemical analysis of the treated PEO1 xenografts. Cisplatin+ Ribociclib-treated tumors demonstrated large acellular regions (Figure 6Di–ii). Immunohistochemical analysis of PEO1 tumors demonstrated a clear decrease in both p-Rb and Ki67 (Figure 6Di–iii) in Ribociclib-treated tumors, indicating on-target activity and efficacy. The greatest decrease in pRb was observed in tumors treated with cisplatin and Ribociclib.

## DISCUSSION

We investigated the effects of Ribociclib as combination and maintenance therapy for high grade serous ovarian cancer (HGSOC). Given that multiple previous reports have shown dysregulated cell cycle gene expression within the known CDKN2A/Cyclin D1-CDK4-CDK6/Rb axis, CDK4/6 inhibition represents a promising approach in ovarian cancer. The tumor suppressor CDKN2A has been shown to be dysregulated through multiple mechanisms, including promoter methylation in 40% of cases in one series [27] and homozygous deletions in 18% of another series [28]. Abnormal CDK4 expression was found in 14–16% of patients in one series and did not differ by tumor stage [29], consistent with a 14% aberrant CDK4 expression rate found through Northern blot analysis in another study [30]. Cyclin D1 was found to be overexpressed in 19% of ovarian tumors in one study, which was correlated with poor prognosis [31]. Rates of RB loss or aberrant expression vary widely, and have ranged from 8–78%, (reviewed in [20]). In our study, mutational analysis of TCGA ovarian cancer data has shown that a significant percentage of patients have mutations or dysregulated expression of CDKN2A, CDK4, CDK6, or CCND1 that would likely make them good candidates for CDK4/6 inhibitor therapy (Figure 1A). However, 17% of ovarian cancer patients in the TCGA database also have homozygous deletions or significantly downregulated RB1; these patients are less likely to receive significant benefit from CDK4/6 inhibitor therapy. Consistent with this mechanism, previous reports have shown that CDKN2A-low/RB1-proficient ovarian cancer cells were most responsive to CDK4/6 inhibition [14] and that RB1 loss was a mechanism of resistance to CDK4/6 inhibition. The correlations between mutational status and response to CDK4/6 inhibition are also clear in breast cancer, where downregulation of CDKN2A and amplification of CDK4 or CDK6 were correlated with sensitivity to CDK4/6 inhibition [7]. This is also concordant with our data showing that ovarian cancer cell lines carrying RB deletions are insensitive to Ribociclib as single-agent therapy.

Several studies have reported that CDK4/6 inhibition can induce cellular senescence [32–34]. Traditionally, senescence is considered to be irreversible exit from the cell cycle into the G0 phase with expression of a suite of senescence-associated secretory markers (reviewed in [23]). Through these secretory factors, senescent cells can promote malignant progression without actively dividing. Despite the traditional association of senescence with the G0 phase, one known mechanism of senescence begins with the tumor suppressor p16^Ink4a^ (CDKN2A), which inhibits Rb inactivation by CDK4 and CDK6, leading to failure to transition from G1 phase into S phase [35, 36]. Therefore, it is possible that CDK4/6 inhibition by Ribociclib could lead to replicative senescence. In our study, Ribociclib treatment was associated with a pseudo-senescent-phenotype *in vitro*; cells showed strong induction of SAβG with partial mRNA induction of some known senescence associated secretory proteins. However, cells continued to proliferate after drug washout, even after a long exposure. Indeed, the majority of cells could proliferate even in the presence of high-dose Ribociclib, albeit more slowly. Based on our data, cell cycle retardation delays tumor growth and can serve as an effective treatment. Given that cells do not truly senesce, continuous therapy will be necessary. However, given the pro-inflammatory and pro-tumorigenic nature of senescence-associated secretory proteins, further investigation is required to better characterize this pseudo-senescent state.

Interestingly, we have observed significant synergy (CI 0.2–0.4) between Ribociclib and cisplatin. While Ribociclib alone retards but does not completely block cell proliferation, the combination of concurrent and maintenance Ribociclib with and after cisplatin *in vitro* arrested cell growth. This was associated with a prolonged arrest of cells in G2/M phase (Figure 4A-C). We hypothesize that this arrest may be related to the known functions of CDKs in the DNA damage response (reviewed in [37, 38]), which is essential for response to and recovery from cisplatin exposure [39]. In particular, cells can become sensitized to cisplatin after ATR depletion [40]; a recent report shows that CDK6 regulates transcription of ATR, and that CDK6 inhibition therefore sensitizes epithelial ovarian cancer cells to death from cisplatin due to an impaired DNA damage response [41]. We found that Ribociclib treatment was associated with a decrease in p-Chk1 in all tested ovarian cancer cells and in ATR in the Hey1 cell line. ATR or Chk-1 mediated dysregulation of the DNA damage response may help to explain the prolonged growth arrest seen with the combination of Ribociclib and cisplatin.

We observed that Ribociclib significantly delayed tumor growth in *in vivo* xenograft experiments when used as a single agent and after cisplatin treatment. Interestingly, Ribociclib therapy was as effective as cytotoxic cisplatin therapy *in vivo* in platinum-sensitive cells, and it had significant activity in platinum-resistant cells. In platinum-sensitive cells, cisplatin + Ribociclib followed by Ribociclib maintenance therapy was not superior to cisplatin alone followed by maintenance therapy. This may be partly because tumor volume measurements were misleading due to large regions of acellular tissues. Previous studies have suggested that concurrent CDK4/6 inhibition with platinum may decrease chemotherapeutic effectiveness [14] as it decreases the cell cycling rate. However, we find that the timing of drug administration is critical, with pre-administration of Ribociclib increasing resistance to cisplatin, but concurrent therapy enhancing efficacy. Given the competing roles of Ribociclib during initial cisplatin therapy in the cytostatic G1-arrest response vs. recovery after cisplatin and the DNA damage response, further studies are necessary to investigate this balance.

In conclusion, CDK4/6 inhibition with Ribociclib showed significant activity against both platinum-sensitive and platinum-resistant cell lines both *in vitro* and *in vivo*. This drug shows significant combinatorial effects with cisplatin, resulting in prolonged times to cellular recovery *in vitro* and restriction of tumor growth *in vivo*. Further research regarding specific mechanisms by which this drug combination affects cell cycling and the DNA damage response as well as clinical impacts is required.

## MATERIALS AND METHODS

### Cell lines

The A2780 cell line (Rb^WT^; platinum-sensitive) was obtained from Dr. Susan Murphy at Duke and was used at passages 12–14. COV504 (Rb^WT^; platinum-sensitive) and OVSAHO (Rb^null^; platinum-sensitive) lines were obtained from Dr. Deborah Marsh at the University of Sydney and were used from passages 5–10. The COV362 (Rb^null^; platinum-sensitive) line was obtained from ATCC and used from passages 6–12. The PEO1 cell line (Rb^WT^; platinum-sensitive) was purchased from Sigma-Aldrich in 12/2016, which uses STR profiling for cell line authentication. The Hey1 cell line (Rb^WT^; platinum-resistant) was obtained from Rebecca Liu at the University of Michigan. The A2780, COV504, Hey1, OVSAHO, and COV362 lines underwent STR profiling in 2/2017 with the American Type Culture Collection (ATCC) for validation; the PEO1 line was not profiled as it had been purchased two months previously. In 2016, the A2780, Hey1, and OVSAHO cell lines tested positive for mycoplasma and were successfully treated with the MycoZap-5 kit (Lonza) and monitored every six months using the MycoAlert detection kit (Lonza) with no subsequent evidence of infection. Hey1, A2780, and COV504 lines were cultured in RPMI-1640 media with 10% FBS and 1% Penicillin/Streptomycin at 37° C and 5% CO2. OVSAHO, PEO1, and COV362 lines were cultured in DMEM with 10% FBS and 1% Penicillin/Streptomycin at 37° C and 5% CO_2_.

### Cell cycle analysis

Hey1 and COV362 cells were grown in 6-well plates in triplicate and treated for 72 hours with 0, 250 nM, 1 uM, or 3 uM Ribociclib (initially purchased from Selleckchem, later generously provided by Novartis) for three days. Cells were then harvested, fixed dropwise in 70% ethanol, and incubated with 0.1 ug/mL RNAse for 1h at 37° F. 1 ug/mL propidium iodide was added and then cells were run on the BD Accuri C6 flow cytometer (Becton Dickinson) and analyzed with FlowJo Version 10. 10,000 events were used for each sample.

### Senescence analysis

Hey1 cells were grown in 6-well dishes in triplicate and treated for three days with 0, 250 nM, 1 uM, or 3 uM Ribociclib. Each well was stained overnight for senescence-associated β-galactosidase with the Senescence β-galactosidase Assay Kit (Cell Signaling) according to the manufacturer’s instructions. Images were quantified as described in the Statistics section.

### Western blots

Western blots were performed as previously described [42]. Briefly, Hey1, COV504, or PEO1 cells were cultured with various concentrations of Ribociclib (clinical grade provided by Novartis) and cisplatin (clinical grade purchased from the University of Michigan Pharmacy) for 3 days, lysed in RIPA buffer (Pierce) with complete protease inhibitor (Roche), and quantified by Bradford assay (Pierce) per the manufacturer’s instructions. Then, 100 ug of protein were loaded onto a 4–12% NuPAGE SDS gel (Thermo Fisher) and transferred to a PVDF membrane (Thermo Fisher). Membranes were incubated overnight with 1:1000 anti-RB, 1:1000 anti-pRB-S807/811, 1:1000 anti-pCHK1, or 1:1000 anti-pATR (all from Cell Signaling) at room temperature and then washed and incubated for 1h with 1:10,000 anti-mouse HRP or anti-rabbit HRP (Cell Signaling). Visualization was performed with ECL Plus Western Blotting Substrate (Pierce). Densitometry and quantification were subsequently performed with ImageJ.

### qRT-PCR

Hey1 cells were grown in 6-well dishes and treated for three days with 0, 250 nM, 1 uM, or 3 uM Ribociclib. Total RNA was extracted with an RNeasy Mini Kit (Qiagen) and quantified with a Nanodrop 1000 (Thermo Scientific). 1 ug RNA was converted to cDNA with a SuperScript III Reverse Transcriptase cDNA Kit (Life Technologies) per the manufacturer’s instructions, and 10 ng of cDNA was used for each reaction. qRT-PCR was performed for 40 cycles using SYBR dye (Applied Biosystems) as recommended by the manufacturer, with primers at 100 nM concentrations each.

Primers for senescence-associated qRT-PCR genes are as follows: CSF2, F-5′-GCTGTCTACGTCGG GATGC-3′, R-5′- GACCATGCGATCCACCTCTC-3′; IL 1A, F-5′- TGGTAGTAGCAACCAACGGGA-3′, R-5′- ACTTTGATTGAGGGCGTCATTC-3′; IL6, F-5′- ACT CACCTCTTCAGAACGAATTG-3′, R-5′- CCATCTTT GGAAGGTTCAGGTTG-3′; ANG F-5′- AGCGCC GAAGTCCAGAAAAC-3′, R-5′- TACTCTCACGACAGT TGCCAT-3′; HRG F-5′- CGGTGTCCATGCCTTCCAT-3′, R-5′- GCGAGTTTCTTAACAGGCTCT-3′; and SERP INB1 F-5′- TTCCTGGCGTTGAGTGAGAAC-3′, R-5′- CTGCCGTGTTACCTCTGGTC-3′. Melt curves were performed to ensure a uniform product, and expression was then normalized to B-Actin with the ΔΔCT method.

### MTT assays

2,000 Hey1 cells were plated into each well of a 96-well plate and treated with various combinations of 1 ug/mL cisplatin and 0, 250 nM, 1 uM, and 3 uM Ribociclib for up to five days, as described in Supplementary Figure 2. Then, media was removed and cells were incubated with MTT and SDS using the Vybrant MTT Cell Proliferation Assay Kit (ThermoFisher Scientific) as described in the manufacturer’s protocol. Absorbance was read at 570 nM and was normalized for the impact of Ribociclib on cell proliferation for analysis.

### Recovery assays

20,000 Hey1, PEO1, or COV504 cells were plated in 12-well dishes and treated with various combinations of Ribociclib and cisplatin. Thereafter, cells were counted every 2–3 days in triplicate with trypan blue exclusion and the recovery of cell number was plotted. Experiments were terminated when cells reached confluence.

### Tumor xenograft experiments

All animal experiments were approved by the University of Michigan Institutional Review Board and the University of Michigan Institutional Animal Cases and Use Committee (IACUC). Nod/SCID/Gamma (NSG) mice were raised under SPF conditions with a 12 hr dark/light cycle and ad-libitum chow and drinking water. Mice were injected subcutaneously with 100,000 Hey1 or PEO1 cells on Day 0 of each experiment. Three days later, mice were treated with PBS, vehicle (1% methylcellulose), cisplatin, Ribociclib, or cisplatin + Ribociclib, according to the dosing schedule provided in Figure 6A. Tumors were measured twice a week with calipers and tumor volumes were calculated using the modified ellipsoid formula: volume = (L*W^2^)/2. Tumor weights were collected when mice were sacrificed at the tumor endpoint, which was defined in our IACUC protocol as a tumor burden >2000 mm^3^ per mouse, >10% weight loss, poor health of the animal, or tumor ulceration. Mice were euthanized when they reached any of these tumor endpoints, and growth curves were plotted for each drug or drug combination. Three animals in each group were sacrificed when animals reached 400–500 mm^3^ for IHC analysis of tumors during active treatment.

### IHC

IHC for Ki67 and pRb was performed by the UMCC histology core on tumors harvested at a volume of 400-500 mm^3^ to minimize central tumor necrosis and better define tumor histology. Ki67 staining was performed as previously described [43]. For pRB, formalin-fixed paraffin-embedded sections were cut at 5-micron thickness and rehydrated with water. Heat induced epitope retrieval was performed with FLEX TRS High pH Retrieval buffer (9.0) for 20 minutes for pRB (Ser 807/811, 1:400) (Cell Signaling, D20B12) and Ki-67 (rabbit monoclonal, Cell Marque 1:2:50). The Dako EnVision+ Rabbit or Mouse System, as appropriate, was used for detection per the manufacturer’s protocol. DAB chromagen was then applied for 10 minutes. Slides were counterstained with Harris Hematoxylin for 5 seconds and then dehydrated and coverslipped.

### Statistical analysis

*In vitro* experiments were repeated independently at least three times with triplicate samples in each experiment, unless indicated otherwise. All mouse studies were performed with *n* = 10 tumors per group, based on a final tumor volume of ∼1000 mm^3^ in control animals and an expected standard deviation of 30%. For SABG analysis, five high power fields from three technical replicates in each treatment group were scored. Similarly, for tumor IHC analysis, five high power fields from three sections of three tumors in each group were scored as previously described [43, 44]. Statistical significance for continuous variables was evaluated using a 2-sided student’s *T*-test or one-way ANOVA, as appropriate, with *p*-values < 0.05 denoting significance. Error bars in figures represent standard error of the mean unless denoted otherwise. Synergy analysis was performed using 12 day time points using the Chou-Tataly median effects method [45] and calculated using Compusyn software (http://www.combosyn.com).

## SUPPLEMENTARY MATERIALS FIGURES



## Abbreviations

LEE-011: Ribociclib
CDK: Cyclin Dependent Kinase
HGSOC: high grade serous ovarian cancer
TCGA: The Cancer Genome Atlas

## References

[1] Aletti GD, Gallenberg MM, Cliby WA, Jatoi A, Hartmann LC (2007). Current management strategies for ovarian cancer. Mayo Clin Proc.

[2] Siegel RL, Miller KD, Jemal A (2015). Cancer statistics, 2015. CA Cancer J Clin.

[3] Markman M, Bookman MA (2000). Second-line treatment of ovarian cancer. Oncologist.

[4] Sherr CJ, Beach D, Shapiro GI (2016). Targeting CDK4 and CDK6: from discovery to therapy. Cancer Discov.

[5] Rader J, Russell MR, Hart LS, Nakazawa MS, Belcastro LT, Martinez D, Li Y, Carpenter EL, Attiyeh EF, Diskin SJ, Kim S, Parasuraman S, Caponigro G (2013). Dual CDK4/CDK6 inhibition induces cell cycle arrest and senescence in neuroblastoma. Clin Cancer Res.

[6] Zhang YX, Sicinska E, Czaplinski JT, Remillard SP, Moss S, Wang Y, Brain C, Loo A, Snyder EL, Demetri GD, Kim S, Kung AL, Wagner AJ (2014). Antiproliferative effects of CDK4/6 inhibition in CDK4-amplified human liposarcoma *in vitro* and *in vivo*. Mol Cancer Ther.

[7] Finn RS, Dering J, Conklin D, Kalous O, Cohen DJ, Desai AJ, Ginther C, Atefi M, Chen I, Fowst C, Los G, Slamon DJ (2009). PD 0332991, a selective cyclin D kinase 4/6 inhibitor, preferentially inhibits proliferation of luminal estrogen receptor-positive human breast cancer cell lines *in vitro*. Breast Cancer Res.

[8] Leonard JP, LaCasce AS, Smith MR, Noy A, Chirieac LR, Rodig SJ, Yu JQ, Vallabhajosula S, Schoder H, English P, Neuberg DS, Martin P, Millenson MM (2012). Selective CDK4/6 inhibition with tumor responses by PD0332991 in patients with mantle cell lymphoma. Blood.

[9] Gopalan PK, Pinder MC, Chiappori A, Ivey AM, Gordillo Villegas A, Kaye FJ (2014). A phase II clinical trial of the CDK 4/6 inhibitor palbociclib (PD 0332991) in previously treated, advanced non-small cell lung cancer (NSCLC) patients with inactivated CDKN2A. J Clin Oncol.

[10] Vaughn DJ, Hwang WT, Lal P, Rosen MA, Gallagher M, O’Dwyer PJ (2015). Phase 2 trial of the cyclin-dependent kinase 4/6 inhibitor palbociclib in patients with retinoblastoma protein-expressing germ cell tumors. Cancer.

[11] Finn RS, Crown JP, Lang I, Boer K, Bondarenko IM, Kulyk SO, Ettl J, Patel R, Pinter T, Schmidt M, Shparyk Y, Thummala AR, Voytko NL (2015). The cyclin-dependent kinase 4/6 inhibitor palbociclib in combination with letrozole versus letrozole alone as first-line treatment of oestrogen receptor-positive, HER2-negative, advanced breast cancer (PALOMA-1/TRIO-18): a randomised phase 2 study. Lancet Oncol.

[12] Finn RS, Martin M, Rugo HS, Jones S, Im SA, Gelmon K, Harbeck N, Lipatov ON, Walshe JM, Moulder S, Gauthier E, Lu DR, Randolph S (2016). Palbociclib and Letrozole in advanced breast cancer. N Engl J Med.

[13] Hortobagyi GN, Stemmer SM, Burris HA, Yap YS, Sonke GS, Paluch-Shimon S, Campone M, Blackwell KL, André F, Winer EP, Janni W, Verma S, Conte P (2016). Ribociclib as first-line therapy for HR-positive, advanced breast cancer. N Engl J Med.

[14] Konecny GE, Winterhoff B, Kolarova T, Qi J, Manivong K, Dering J, Yang G, Chalukya M, Wang HJ, Anderson L, Kalli KR, Finn RS, Ginther C (2011). Expression of p16 and retinoblastoma determines response to CDK4/6 inhibition in ovarian cancer. Clin Cancer Res.

[15] Bonuccelli G, Peiris-Pages M, Ozsvari B, Martinez-Outschoorn UE, Sotgia F, Lisanti MP (2017). Targeting cancer stem cell propagation with palbociclib, a CDK4/6 inhibitor: Telomerase drives tumor cell heterogeneity. Oncotarget.

[16] Taylor-Harding B, Aspuria PJ, Agadjanian H, Cheon DJ, Mizuno T, Greenberg D, Allen JR, Spurka L, Funari V, Spiteri E, Wang Q, Orsulic S, Walsh C (2015). Cyclin E1 and RTK/RAS signaling drive CDK inhibitor resistance via activation of E2F and ETS. Oncotarget.

[17] Patnaik A, Rosen LS, Tolaney SM, Tolcher AW, Goldman JW, Gandhi L, Papadopoulos KP, Beeram M, Rasco DW, Hilton JF, Nasir A, Beckmann RP, Schade AE (2016). Efficacy and safety of abemaciclib, an inhibitor of CDK4 and CDK6, for patients with breast cancer, non-small cell lung cancer, and other solid tumors. Cancer Discov.

[18] DeMichele A, Clark AS, Tan KS, Heitjan DF, Gramlich K, Gallagher M, Lal P, Feldman M, Zhang P, Colameco C, Lewis D, Langer M, Goodman N (2015). CDK 4/6 inhibitor palbociclib (PD0332991) in Rb+ advanced breast cancer: phase II activity, safety, and predictive biomarker assessment. Clin Cancer Res.

[19] Bell D, Berchuck A, Birrer M, Chien J, Cramer DW, Dao F, Dhir R, DiSaia P, Gabra H, Glenn P, Godwin AK, Gross J, Hartmann L, Cancer Genome Atlas Research Network (2011). Integrated genomic analyses of ovarian carcinoma. Nature.

[20] Corney DC, Flesken-Nikitin A, Choi J, Nikitin AY (2008). Role of p53 and Rb in ovarian cancer. Adv Exp Med Biol.

[21] Ruas M, Peters G (1998). The p16INK4a/CDKN2A tumor suppressor and its relatives. Biochim Biophys Acta.

[22] Liu G, Sun Y, Ji P, Li X, Cogdell D, Yang D, Parker Kerrigan BC, Shmulevich I, Chen K, Sood AK, Xue F, Zhang W (2014). MiR-506 suppresses proliferation and induces senescence by directly targeting the CDK4/6-FOXM1 axis in ovarian cancer. J Pathol.

[23] Coppé JP, Desprez PY, Krtolica A, Campisi J (2010). The senescence-associated secretory phenotype: the dark side of tumor suppression. Annu Rev Pathol.

[24] Heinrichs A (2008). Cell division: back and forth. Nat Rev Cancer.

[25] Sorenson CM, Barry MA, Eastman A (1990). Analysis of events associated with cell cycle arrest at G2 phase and cell death induced by cisplatin. J Natl Cancer Inst.

[26] Sorenson CM, Eastman A (1988). Mechanism of cis-diamminedichloroplatinum(II)-induced cytotoxicity: role of G2 arrest and DNA double-strand breaks. Cancer Res.

[27] Katsaros D, Cho W, Singal R, Fracchioli S, Rigault De La Longrais IA, Arisio R, Massobrio M, Smith M, Zheng W, Glass J, Yu H (2004). Methylation of tumor suppressor gene p16 and prognosis of epithelial ovarian cancer. Gynecol Oncol.

[28] Kudoh K, Ichikawa Y, Yoshida S, Hirai M, Kikuchi Y, Nagata I, Miwa M, Uchida K (2002). Inactivation of p16/CDKN2 and p15/MTS2 is associated with prognosis and response to chemotherapy in ovarian cancer. Int J Cancer.

[29] Kusume T, Tsuda H, Kawabata M, Inoue T, Umesaki N, Suzuki T, Yamamoto K (1999). The p16-cyclin D1/CDK4-pRb pathway and clinical outcome in epithelial ovarian cancer. Clin Cancer Res.

[30] Masciullo V, Scambia G, Marone M, Giannitelli C, Ferrandina G, Bellacosa A, Benedetti Panici P, Mancuso S (1997). Altered expression of cyclin D1 and CDK4 genes in ovarian carcinomas. Int J Cancer.

[31] Bali A, O’Brien PM, Edwards LS, Sutherland RL, Hacker NF, Henshall SM (2004). Cyclin D1, p53, and p21Waf1/Cip1 expression is predictive of poor clinical outcome in serous epithelial ovarian cancer. Clin Cancer Res.

[32] Valenzuela CA, Vargas L, Martinez V, Bravo S, Brown NE (2017). Palbociclib-induced autophagy and senescence in gastric cancer cells. Exp Cell Res.

[33] Yoshida A, Diehl JA (2015). CDK4/6 inhibitor: from quiescence to senescence. Oncoscience.

[34] Yoshida A, Lee EK, Diehl JA (2016). Induction of therapeutic senescence in vemurafenib-resistant melanoma by extended inhibition of CDK4/6. Cancer Res.

[35] Alcorta DA, Xiong Y, Phelps D, Hannon G, Beach D, Barrett JC (1996). Involvement of the cyclin-dependent kinase inhibitor p16 (INK4a) in replicative senescence of normal human fibroblasts. Proc Natl Acad Sci USA.

[36] Takahashi A, Ohtani N, Yamakoshi K, Iida S, Tahara H, Nakayama K, Nakayama KI, Ide T, Saya H, Hara E (2006). Mitogenic signalling and the p16INK4a-Rb pathway cooperate to enforce irreversible cellular senescence. Nat Cell Biol.

[37] Johnson N, Shapiro GI (2010). Cyclin-dependent kinases (cdks) and the DNA damage response: rationale for cdk inhibitor–chemotherapy combinations as an anticancer strategy for solid tumors. Expert Opin Ther Targets.

[38] Trovesi C, Manfrini N, Falcettoni M, Longhese MP (2013). Regulation of the DNA damage response by cyclin-dependent kinases. J Mol Biol.

[39] Basu A, Krishnamurthy S (2010). Cellular responses to Cisplatin-induced DNA damage. J Nucleic Acids.

[40] Wagner JM, Karnitz LM (2009). Cisplatin-induced DNA damage activates replication checkpoint signaling components that differentially affect tumor cell survival. Mol Pharmacol.

[41] Dall’Acqua A, Sonego M, Pellizzari I, Pellarin I, Canzonieri V, D’Andrea S, Benevol S, Sorio R, Giorda G, Califano D, Bagnoli M, Militello L, Mezzanzanica D (2017). CDK6 protects epithelial ovarian cancer from platinum-induced death via FOXO3 regulation. EMBO Mol Med.

[42] Coffman LG, Choi YJ, McLean K, Allen BL, di Magliano MP, Buckanovich RJ (2016). Human carcinoma-associated mesenchymal stem cells promote ovarian cancer chemotherapy resistance via a BMP4/HH signaling loop. Oncotarget.

[43] Bai S, Ingram P, Chen YC, Deng N, Pearson A, Niknafs Y, O’Hayer P, Wang Y, Zhang ZY, Boscolo E, Bischoff J, Yoon E, Buckanovich RJ (2016). EGFL6 regulates the asymmetric division, maintenance, and metastasis of ALDH+ ovarian cancer cells. Cancer Res.

[44] Pulaski HL, Spahlinger G, Silva IA, McLean K, Kueck AS, Reynolds RK, Coukos G, Conejo-Garcia JR, Buckanovich RJ (2009). Identifying alemtuzumab as an anti-myeloid cell antiangiogenic therapy for the treatment of ovarian cancer. J Transl Med.

[45] Chou TC, Talalay P (1983). Analysis of combined drug effects: a new look at a very old problem. Trends Pharmacol Sci.

