# Supplementary material for: CDK4/6 inhibition as maintenance and combination therapy for high grade serous ovarian cancer
Source: Oncotarget. 2018 Feb 26;9(21):15658–72. doi: 10.18632/oncotarget.24585 (PMC5884655; doi:10.18632/oncotarget.24585)
Supplement: Supplementary file 1 [file oncotarget-09-15658-s001.pdf]

CDK4/6 inhibition as maintenance and combination therapy for high grade serous ovarian cancer

SUPPLEMENTARY MATERIALS

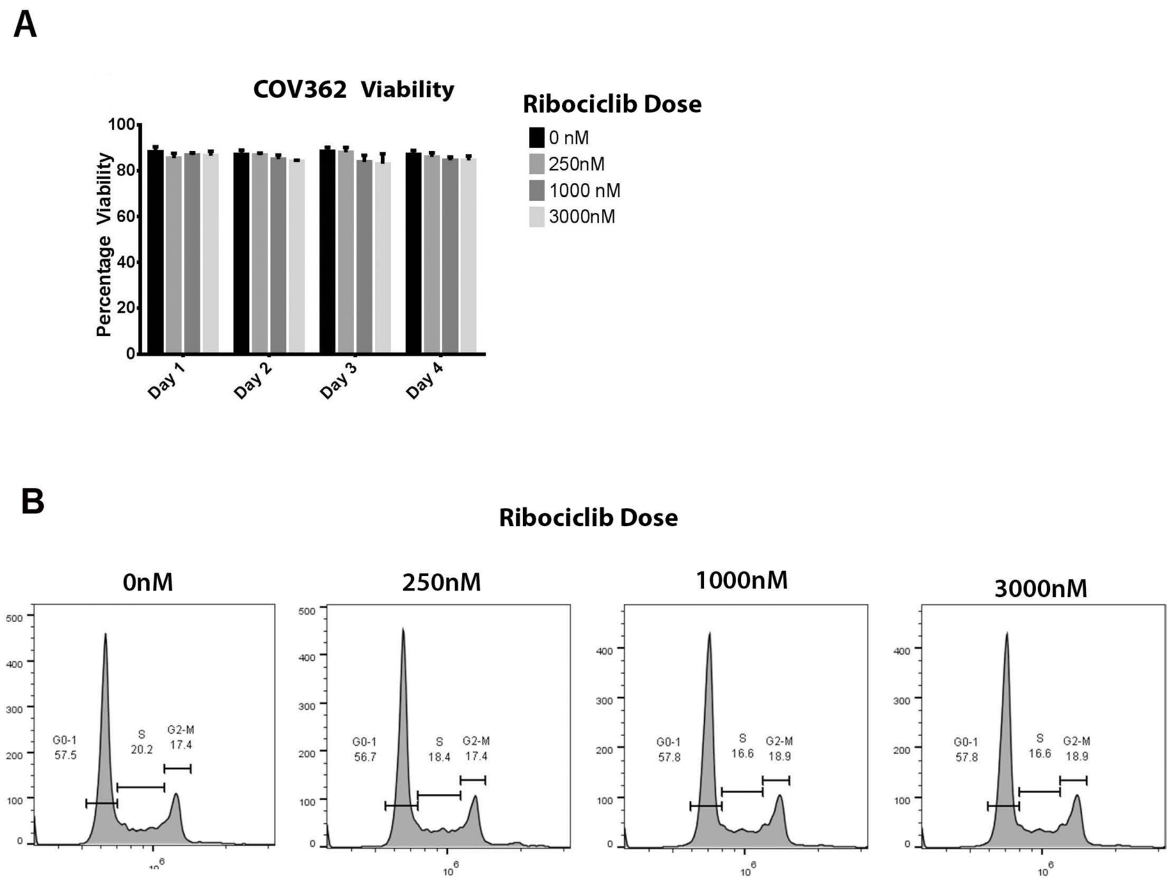

Supplementary Figure 1: Ribociclib has no impact on viability or cell cycling in RB^{null} COV362 cells. (**A**) Total cell viability with increasing doses of Ribociclib in the RB^{null} COV362 line. (**B**) Cell cycle profile of COV362 cells treated with the indicated doses of Ribociclib.

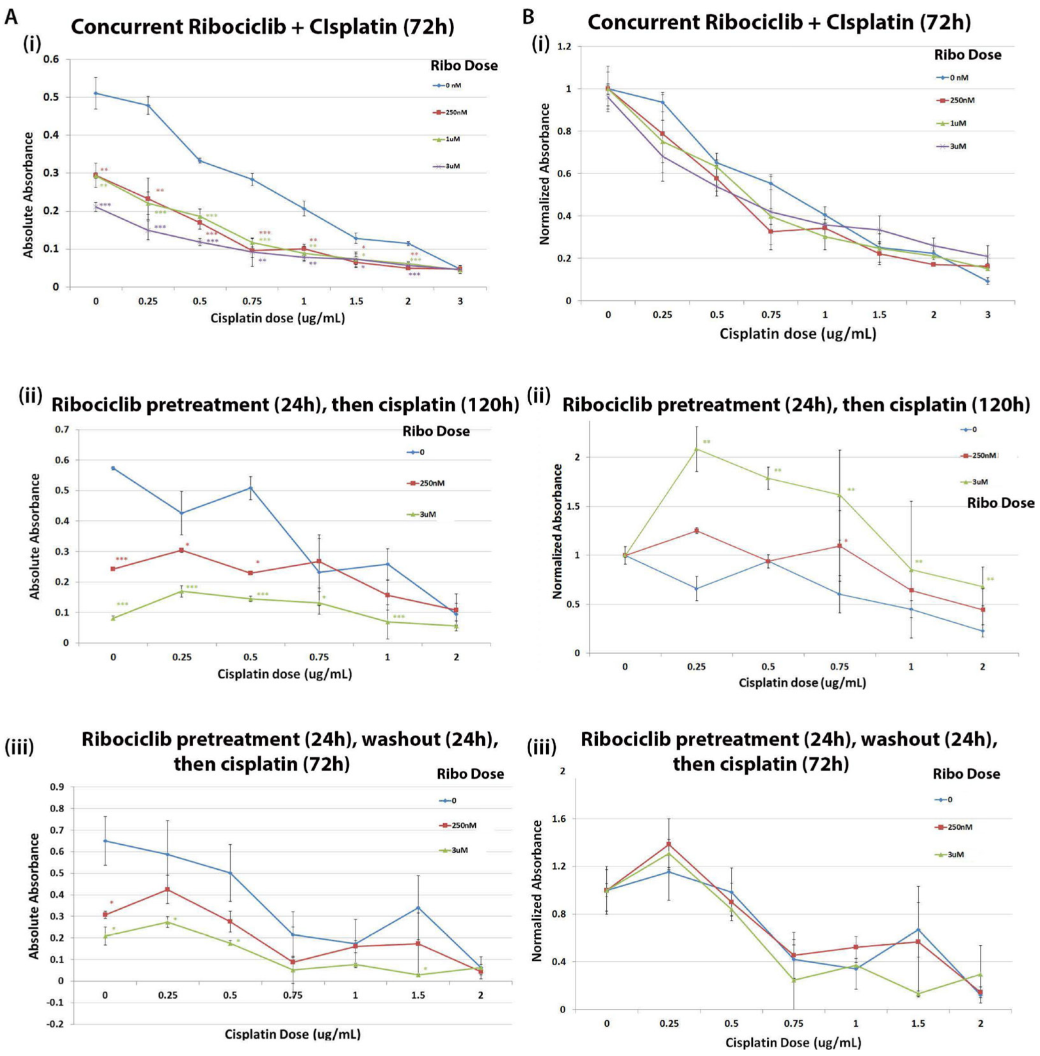

Supplementary Figure 2: Impact of Ribociclib and cisplatin on total cell viability. Absolute (**A**) and normalized (**B**) MTT absorbance as a measure of viability in Hey1 ovarian cancer cells treated with various combinations of Ribociclib and cisplatin. To correct for cell number, (B) is normalized to absorbance at the indicated Ribociclib dose only, without cisplatin. $^{*}p < 0.05$; $^{**}p < 0.005$; $^{***}p < 0.0005$, compared to the cisplatin-only control (shown in blue).

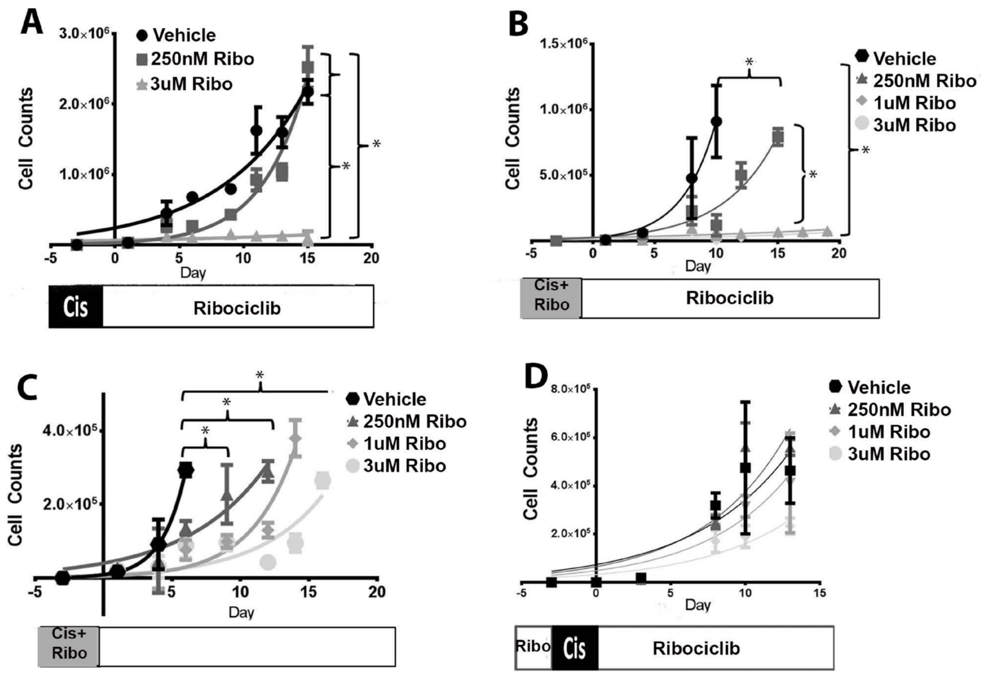

Supplementary Figure 3: Impact of concurrent cisplatin and Ribociclib with and without maintenance therapy in Hey1 cells. Absolute cell numbers over time in Hey1 ovarian cancer cells (**A**) treated with maintenance therapy at the indicated dose of Ribociclib after 72 h of treatment with 1ug/mL cisplatin, (**B**) treated with concurrent cisplatin+Ribociclib followed by the indicated dose of Ribociclib maintenance therapy, (**C**) treated with concurrent cisplatin and the indicated dose of Ribociclib with no subsequent maintenance therapy, or (**D**) pretreated with 48 hrs of Ribociclib, followed by 72 h treatment with 1 ug/mL cisplatin, followed by maintenance treatment with the indicated dose of Ribociclib. All samples were analyzed in triplicate with each experiment replicated at least once. $^{*}p < 0.05$, $^{**}p < 0.01$, $^{***}p < 0.001$.
